# Efficacy and Safety of BTKis in Central Nervous System Lymphoma: A Systematic Review and Meta-Analysis

**DOI:** 10.3390/cancers16050860

**Published:** 2024-02-21

**Authors:** Yan Zhang, Jingjing Ye, Hao Chen, Daobin Zhou, Chunyan Ji

**Affiliations:** 1 Department of Hematology, Peking Union Medical College Hospital, Beijing 100730, China; 2 Department of Hematology, Qilu Hospital of Shandong University, Jinan 250012, China; 3 College of Acupuncture and Chinese Tuina, Nanjing University of Chinese Medicine, Nanjing 210023, China

**Keywords:** central nervous system lymphoma, Bruton tyrosine kinase inhibitors, efficacy, safety, meta-analysis

## Abstract

**Simple Summary:**

Central nervous system lymphoma (CNSL) is a highly aggressive form of lymphoma. However, treatments such as chemotherapy and radiotherapy may be intolerable for older or specific patients due to the associated toxicity and severe neurotoxicity. Some studies have shown that Bruton tyrosine kinase inhibitors (BTKis) are effective for CNSL. However, most of these studies were small-sample studies, so the efficacy and safety of BTKis in CNSL are not yet clearly defined. Furthermore, the existing reviews have mainly focused on patients with relapsed/refractory CNSL or primary CNSL, and there have been fewer studies on second-generation BTKis, with a lack of reviews on the evidence of BTKis for CNSL. Therefore, this systematic review aims to comprehensively and systematically elucidate the efficacy and safety of BTKis in the treatment of CNSL. The results indicate that BTKis are effective not only for relapsed/refractory cases but also for newly diagnosed CNSL, with an acceptable safety profile. However, high-quality clinical trials are still needed in the future.

**Abstract:**

Background: This systematic review and meta-analysis aimed to evaluate the efficacy and safety of Bruton tyrosine kinase inhibitors (BTKis) for central nervous system lymphoma (CNSL). Methods: A systematic review was carried out to identify relevant studies from the PubMed, Embase, Cochrane Library, Web of Science, WanFang, CNKI, and CBM databases. The studies included patients with CNSL who received BTKis and reported the overall response (OR), complete remission (CR), and partial response (PR). An overall effect analysis was performed using STATA 15.0. A random-effects model was utilized to calculate the pooled rates, and 95% confidence intervals (CI) were determined for all outcomes. Results: A total of 21 studies involving 368 patients were included in the meta-analysis. For newly diagnosed CNSL, due to the small simple size, we conducted a quantitative description, and the ORR could reach up to 100%. For relapsed/refractory patients, the pooled ORR was 72% (95% CI: 64–80%, I^2^ = 54.89%, *p* = 0.00), with a pooled CR and PR of 43% (95% CI: 33–54%, I^2^ = 65.40%, *p* = 0.00) and 23% (95% CI: 13–35%, I^2^ = 78.05%, *p* = 0.00), respectively. Most adverse events were hematology-related and generally manageable. Conclusion: BTKis showed acceptable efficacy and safety in treating patients with CNSL. However, large and well-designed trials are still required to confirm BTKis as a treatment for CNSL.

## 1. Introduction

Central nervous system lymphoma (CNSL) is an aggressive lymphoma. The 2021 WHO Classification of Tumors of the Central Nervous System includes subtypes of primary diffuse large B-cell lymphoma of the CNS (DLBCL), immunodeficiency-associated CNS lymphoma, lymphomatoid granulomatosis, and intravascular large B-cell lymphoma [1]. Furthermore, CNSL is also divided into primary central nervous system lymphoma (PCNSL) and secondary central nervous system lymphoma (SCNSL). PCNSL is characterized by involvement limited to the brain, spinal cord, cranial nerves, leptomeninges, and vitreo-retina [2,3], and DLBCL accounts for about 80–90% of cases [4]. SCNSL refers to CNS involvement secondary to systemic lymphoma at the time of initial diagnosis or relapse, and it is clinically divided into treatment-naive SCNSL (TN-SCNSL), relapsed isolated CNSL (RI-SCNSL), and relapsed concomitant systemic and CNS disease following treatment for systemic lymphoma (RC-SCNSL) [5]. The guidelines for the treatment of PCNSL mainly include systemic chemotherapy, immunotherapy, intrathecal chemotherapy, radiotherapy, and novel agents. The conventional treatment of RI-SCNSL is basically the same as that of PCNSL, and no guidelines have been formulated at present [6,7]. The backbone of first-line treatments for PCNSL patients is high-dose methotrexate (HD-MTX)-based chemotherapy, with or without other a therapy such as cytarabine, thiotepa, and temozolomide. However, the existing first-line treatments may be unsuitable for older or specific patients, due to related toxicity and severe neurotoxicity. Patients with CNSL usually have a poor prognosis, and most of them may progress to relapse or refractory status. Therefore, the introduction of less toxic but effective targeted agents is needed as an alternative [8,9].

The main hallmark of PCNSL is the activation of nuclear factor kappa-B (NF-κB), while the activation of NF-κB is driven by B cell receptor (BCR) and Toll-like receptor (TLR) signaling pathways [8]. Both healthy and malignant B cells depend on BCR signaling for their growth and survival [10]. Bruton tyrosine kinase inhibitors (BTKis) are important regulators of the BCR pathway, which can participate in the survival and proliferation of malignant B lymphocytes and primarily function by controlling the oncogenic signal transduction downstream of BCR [11,12]. In recent years, BTKis have been suggested as a novel agent for CNSL [6,13]. Ibrutinib, the first BTKi, was granted a breakthrough drug designation by the FDA in 2013 and approved by the FDA for the treatment of patients with mantle-cell lymphoma. In addition, a number of studies have shown that ibrutinib has a good effect on CNSL, especially for relapse/refractory CNSL [14,15].

At present, due to the safety issue of ibrutinib, second-generation BTKis are gradually emerging, such as tirabrutinib, zanubrutinib, acalabrutinib, and orelabrutinib. Currently, ibrutinib, zanubrutinib, acalabrutinib, and orelabrutinib have been marketed in China. Similar to the first-generation BTKis, second-generation BTKis inhibit BCR signaling by covalently binding to BTK C481 and inhibiting BTK kinase, thereby inhibiting the growth and metastasis of malignant proliferating B cells. But they are more selective than ibrutinib [16] and can induce higher concentrations in the cerebrospinal fluid (CSF). Ibrutinib is less selective and can also target EGFR, BMX, and TEC in addition to BTK. However, second-generation BTKis have improved kinase selectivity overall, especially orelabrutinib. Compared to other second-generation BTKis, it has higher kinase selectivity and only targets BTK with >90% inhibition [17]. Narita et al. found that 480 mg (qd) of tirabrutinib resulted in longer PFS (11.1 vs. 2.1 months), higher ORR (100% vs. 60%), and higher CSF trough concentrations (16.3 ± 7.71 vs. 2.19 ± 0.476 ng/mL) than 320 mg (qd) [18]. This suggests that the choice of therapeutic drugs with good CSF penetration may lead to better efficacy to patients with CNSL. Meanwhile, the mean CSF concentrations were higher for tirabrutinib (320 mg, qd, 2.19 ng/mL; 480 mg, qd, 14.0 ng/mL) and zanubrutinib (160 mg, bid, 2.94 ng/mL) compared to ibrutinib (560 mg, qd, 0.62 ng/mL; 700 mg, qd, 0.87 ng/mL; 840 mg, qd, 0.59 ng/mL) [19]. Furthermore, as a novel, small-molecule, selective irreversible BTKi, orelabrutinib (150 mg, qd) has shown better blood–brain barrier permeability and could induce a median CSF concentration as high as 28.7 ng/mL [20]. Some evidence has revealed that orelabrutinib-containing treatment was well-tolerated and provided incremental benefits in Chinese CNSL patients [21,22].

In recent years, BTKis have been increasingly reported in CNSL, but they are more limited to small-sample studies. Furthermore, the existing reviews have mainly focused on the efficacy and safety of ibrutinib for the treatment of relapsed/refractory CNSL patients or PCNSL patients [14,15], and there is a lack of systematic studies regarding BTKis in the management of CNSL. Therefore, the aim of this systematic review was to elucidate the efficacy and safety of various BTKis in the treatment of CNSL more comprehensively and systematically.

## 2. Materials and Methods

This systematic review and meta-analysis were conducted according to the Preferred Reporting Items for Systematic Review and Meta-analysis (PRISMA) statement [23]. A detailed PRISMA checklist is attached in Supplementary Materials S1. This study has not been registered in PROSPERO.

### 2.1. Search Strategy

A systematic search was performed in the Pubmed, Web of Science, Embase, Cochrane Library, China National Knowledge Infrastructure (CNKI), WanFang, and Chinese Biomedical Literature (CBM) databases for the relevant studies before 8 June 2023. The search terms included “central nervous system lymphoma”, “CNS lymphoma”, “CNSL”, “Bruton tyrosine kinase inhibitors”, “Bruton tyrosine kinase”, “BTK inhibitor”, “BTKi”, “ibrutinib”, “acalabrutinib”, “zanubrutinib”, “orelabrutinib”, “pirtobrutinib”, and “tirabrutinib”. The detailed search strategy is shown in Supplementary Materials S2. In addition, the current clinical guidelines of CNSL and the relevant systematic review and meta-analysis were also identified to check the reference lists for relevant studies.

### 2.2. Study Selection and Data Extraction

The inclusion criteria were as follows: (1) Prospective studies and retrospective studies. (2) Patients of any age who were diagnosed with CNSL, irrespective of primary or secondary. PCNSL refers to lymphoma that is limited to the brain, leptomeninges, spinal cord, and eyes at primary diagnosis and without involvement outside the CNS. For SCNSL, only patients with RI-SCNSL were included, which refers to CNS relapse without recurrent systemic lymphoma because the conventional treatment of RI-SCNSL is basically the same as that of PCNSL. (3) BTKis should be used as monotherapy or combination therapy for CNSL patients. (4) The primary outcome was the tumor response, which included the overall response rate (ORR), complete response (CR), and partial response (PR) according to the International Primary CNSL Collaborative Group criteria. Median progression-free survival (mPFS), median overall survival (mOS), and adverse events (AEs) were also considered as the main outcomes in this meta-analysis. PFS was defined as time from initiation of BTKis to disease progression, death from any cause. OS was defined as time from initiation of BTKis to death from any cause.

The exclusion criteria were as follows: (1) Duplicated studies. (2) The sample size was smaller than 2 cases. (3) Studies that were published in neither English nor Chinese. (4) Studies from conference abstracts were also excluded because they lacked peer review.

Two reviewers screened the title, abstract, and full text of the identified studies independently, and any disagreements were resolved by the third reviewer. A pre-designed electronic form was created for the data extraction from each study, which included the authors, year of publication, number of patients, gender, age, study design, detail of the therapy, follow-up period, and outcomes.

### 2.3. Quality Assessment

We implemented the Cochrane Collaboration tool to assess the risk of bias for the included randomized control trials (RCTs). The retrospective studies without a comparison group were evaluated using the JBI Critical Appraisal Checklist of Case Series [24].

### 2.4. Statistical Analysis

The pooling procedure for the included studies was carried out in STATA 15.0 using the “Meta prop” command [25]. The effect size of all pooled results was presented with 95% confidence intervals (CI). The heterogeneity of the studies was assessed using Cochran’s Q test and I^2^ statistics. The random-effects model was used for pooling analysis in this study due to the potential heterogeneity among the included studies. A two-sided *p* < 0.05 was considered statistically significant.

## 3. Results

### 3.1. Study Selection and Characteristics

**Figure 1:**
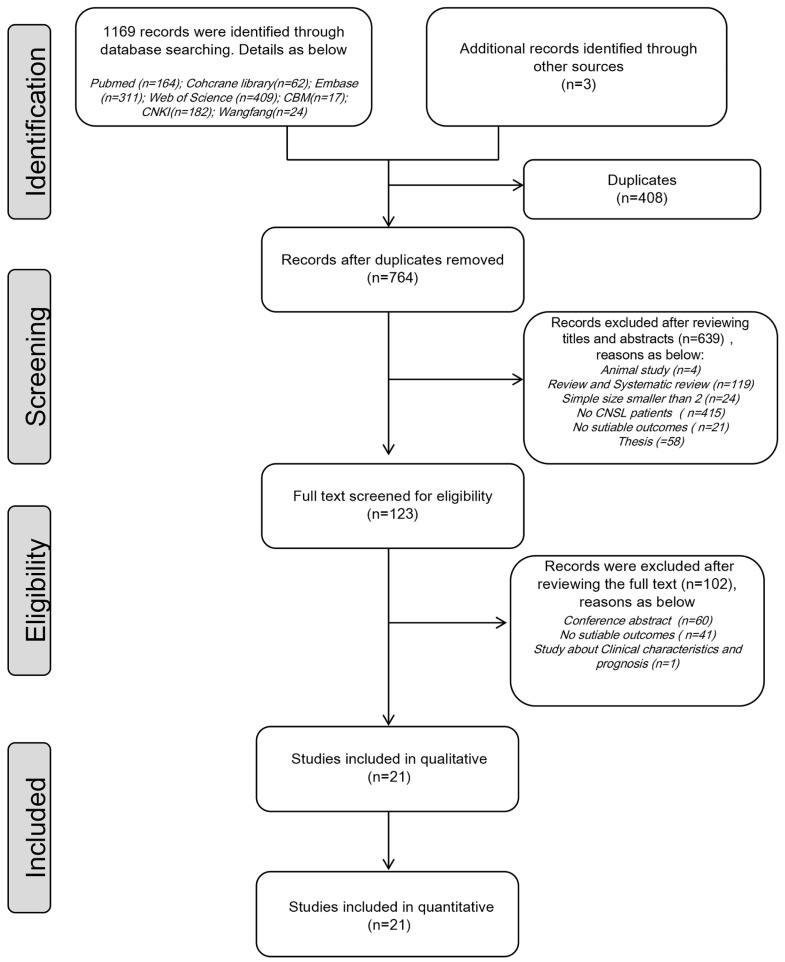
Flow diagram of study selection.

A total of 761 relevant studies were identified in the initial electronic search, and 3 additional relevant studies from the existing guidelines were conformed during the checking of the reference lists of the systematic reviews and guidelines. After screening the titles, abstracts, and the full texts, studies were excluded for different reasons. A total of 21 studies were included in the final analysis [9,18,19,21,22,26,27,28,29,30,31,32,33,34,35,36,37,38,39,40,41]. Figure 1 shows the literature evaluation and identification process. In this meta-analysis, 15 retrospective studies [9,19,21,22,26,28,29,31,33,34,36,37,38,40,41] and 6 prospective studies [18,27,30,32,35,39] were included. Together, 7 (7/21) studies [9,19,22,33,35,38,39] involved a total of 35 patients with newly diagnosed CNSL, while 18 (18/21) [18,19,21,22,26,27,28,29,30,31,32,33,34,35,36,39,40,41] studies involved 333 patients with relapsed/refractory CNSL. The characteristics of each study included are summarized in Table 1.

### 3.2. Quality Assessment of the Included Studies

All of the included studies were assessed by the JBI Critical Appraisal Checklist of Case Series. Most of the items were evaluated as “Yes” except Q9 (Table 2), which presented a moderate quality of included studies.

### 3.3. Efficacy

#### 3.3.1. Tumor Response

**Figure 2:**
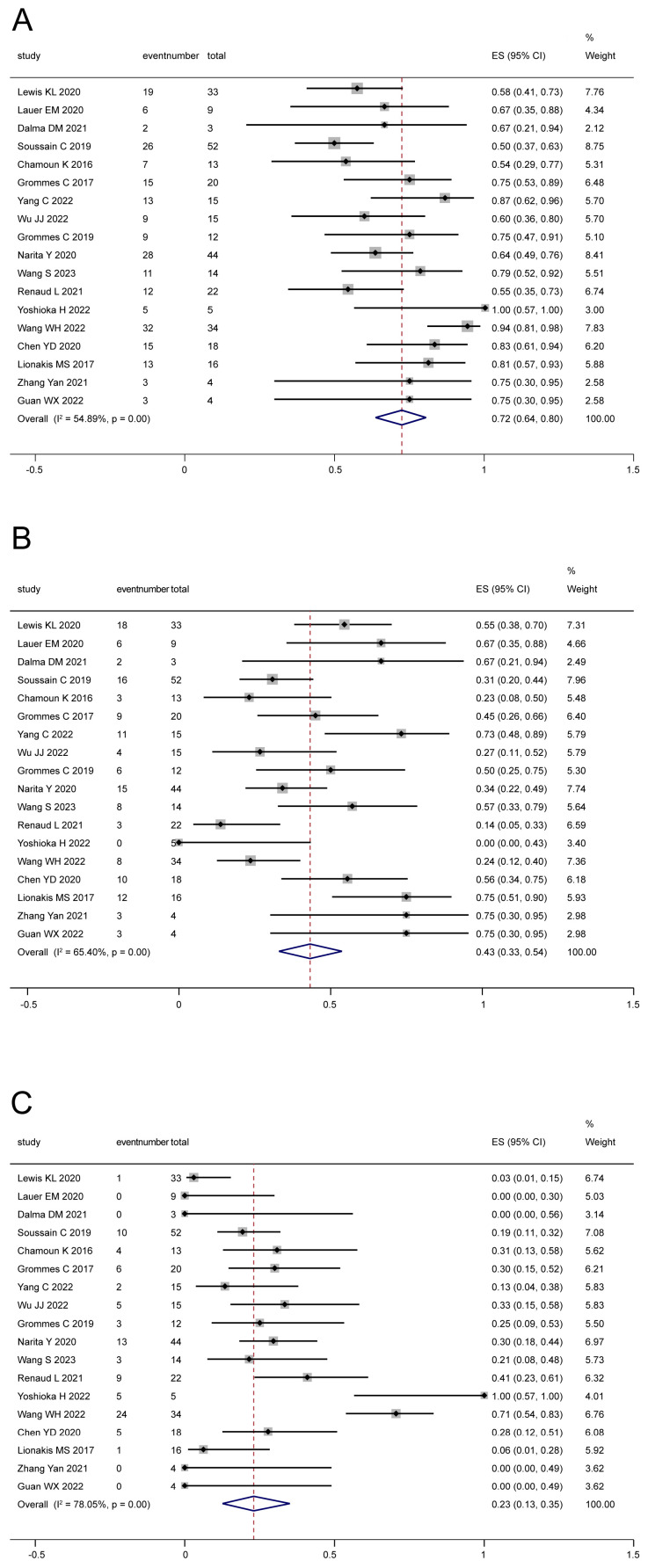
The pooled ORR (**A**), CR (**B**), and PR (**C**) for relapsed/refractory patients [18,19,21,22,26,27,28,29,30,31,32,33,34,35,36,39,40,41].

For newly diagnosed CNSL, 7 studies [9,19,22,33,35,38,39] reported the ORR, CR, and PR. We did not perform a pooled analysis due to the small simple size. A quantitative description is shown in Table 3. The highest ORR and CR were both up to 100%. For relapsed/refractory CNSL patients, 18 studies [18,19,21,22,26,27,28,29,30,31,32,33,34,35,36,39,40,41] reported the ORR, CR and PR. The pooled ORR (Figure 2A) was 72% (95% CI: 64–80%, I^2^ = 54.89%, *p* = 0.00), and the pooled CR (Figure 2B) and PR (Figure 2C) were 43% (95% CI: 33–54%, I^2^ = 65.40%, *p* = 0.00) and 23% (95% CI: 13–35%, I^2^ = 78.05%, *p* = 0.00), respectively.

**Figure 3:**
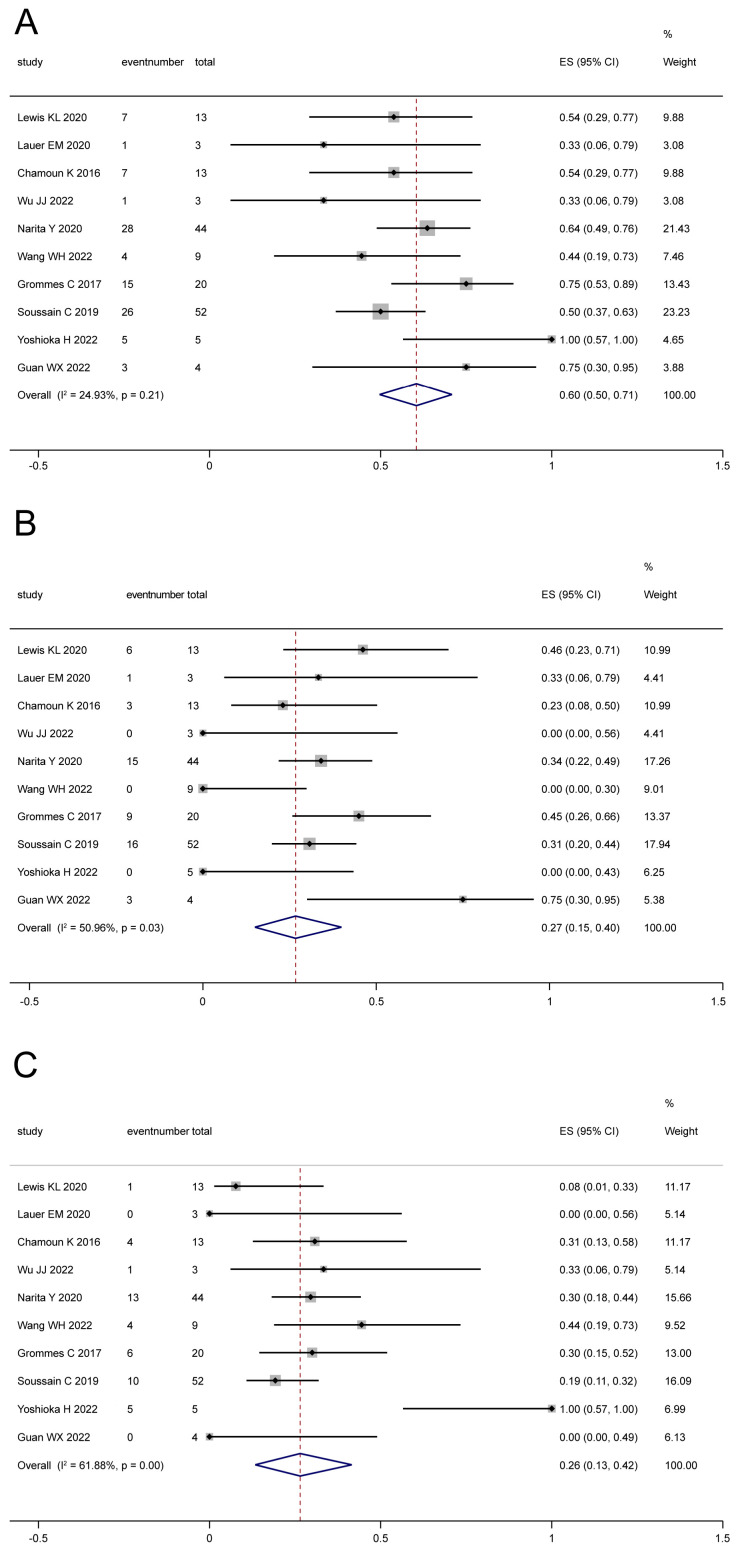
The pooled ORR (**A**), CR (**B**), and PR (**C**) with BTKi monotherapy for relapsed/refractory patients [18,22,26,27,28,30,31,32,39,41].

**Figure 4:**
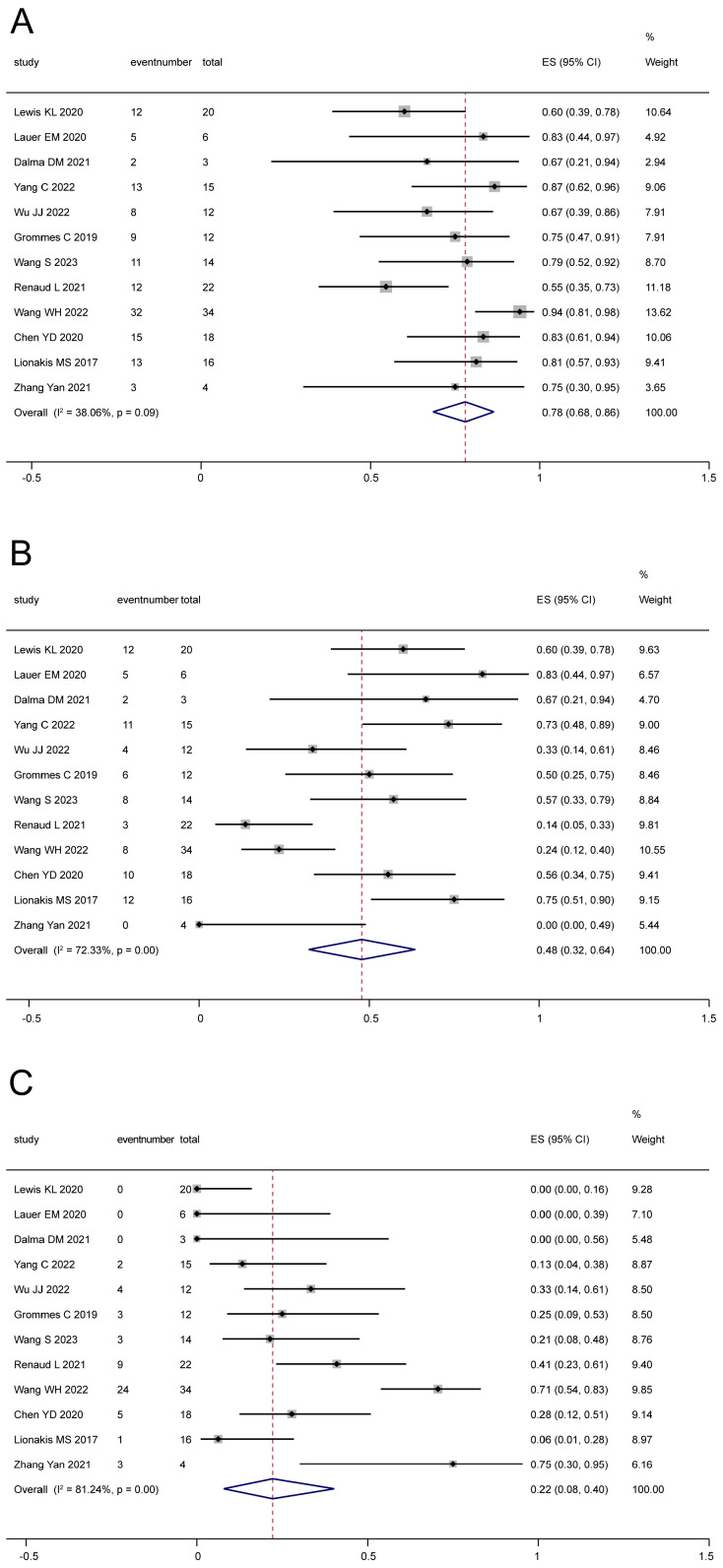
The pooled ORR (**A**), CR (**B**), and PR (**C**) with BTKi combination therapy for relapsed/refractory patients [19,21,22,27,28,29,33,34,35,36,40,41].

For relapsed/refractory CNSL, BTKi monotherapy was provided in 10 studies [18,22,26,27,28,30,31,32,39,41], which involved 166 patients in total. Based on the analysis, the pooled ORR (Figure 3A) was 60% (95% CI: 50–71%, I^2^ = 24.93%, *p* = 0.21), and the pooled CR (Figure 3B) and PR (Figure 3C) were 27% (95% CI: 15–40%, I^2^ = 50.96%, *p* = 0.03) and 26% (95% CI: 13–42%, I^2^ = 61.88%, *p* = 0.00), respectively. Meanwhile, BTKi-based regimes were reported in 12 studies [19,21,22,27,28,29,33,34,35,36,40,41], with 176 patients. BKTis were used as a combination therapy with chemotherapy, radiotherapy, or other therapies. The pooled ORR (Figure 4A) was 78% (95% CI: 68–86%, I^2^ = 38.06%, *p* = 0.09), and the pooled CR (Figure 4B) and PR (Figure 4C) were 48% (95% CI: 32–64%, I^2^ = 72.33%, *p* = 0.00) and 22% (95% CI: 8–40%, I^2^ = 81.24%, *p* = 0.00), respectively.

#### 3.3.2. Survival

**Figure 5:**
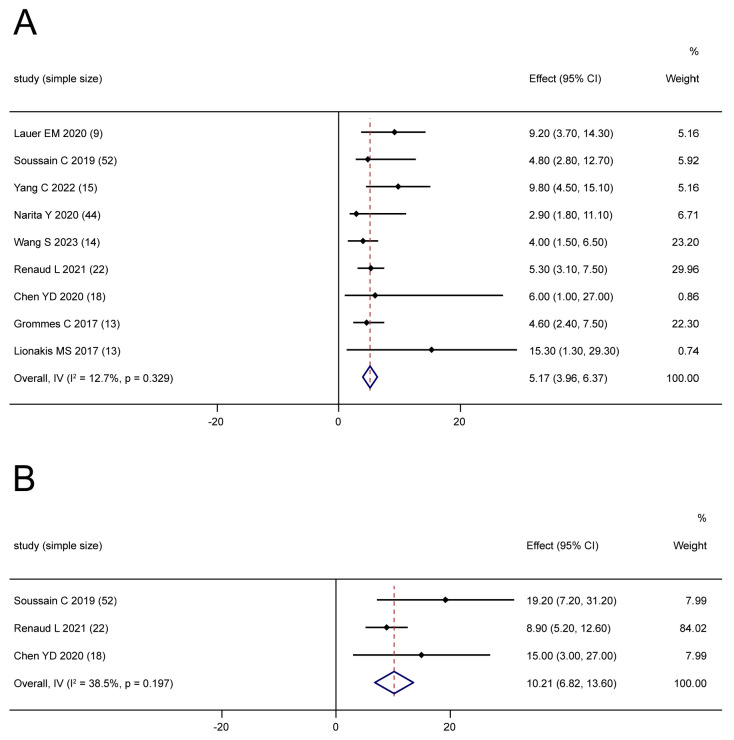
The pooled mPFS (**A**) and mOS (**B**) for relapsed/refractory patients [18,21,28,30,32,33,34,36,40].

Nine [18,21,28,30,32,33,34,36,40] and three [30,36,40] studies reported the mPFS and mOS of relapsed/refractory patients treated by BTKis, respectively. The pooled mPFS was 5.17 months (95% CI: 3.96–6.37, I^2^ = 12.7%, *p* = 0.329, Figure 5A), and the pooled mOS was 10.21 months (95% CI: 6.82–13.60, I^2^ = 38.5%, *p* = 0.197, Figure 5B).

Based on the subgroup analysis, the mPFS of combination therapies was reported in seven studies [21,28,32,33,34,36,40], which involved 104 patients, and the pooled result was 5.36 months (95% CI: 4.08–6.65, I^2^ = 26.3%, *p* = 0.228). The mPFS of monotherapy [18,30] was 4.8 months (95% CI: 2.80, 12.7) and 2.9 months (95% CI: 1.80, 11.10) in two small studies with high heterogeneity.

### 3.4. Adverse Events (AEs)

A total of 12 studies [18,19,21,22,30,32,34,35,36,38,40,41] have reported 969 AEs in any grade. The main AEs in BTKi treatment were associated with hematological AEs, including neutropenia, anemia, thrombocytopenia, leukopenia, lymphopenia, and febrile neutropenia (details in Supplementary Materials S3). The pooled rates of grade 3–5 hematological AEs, such as thrombocytopenia, neutropenia, anemia, leukopenia, lymphopenia, and febrile neutropenia were 9% (2–18%), 12% (4–22%), 12% (5–20%), 10% (2–20%), 19% (0–56%), and 4% (0–19%), respectively. Meanwhile, the subgroup analysis showed the second-generation BTKi treatment resulted in a numerically lower incidence rate of grade 3–5 AEs compared to ibrutinib treatment (Table 4).

## 4. Discussion

In recent guidelines, BTKis were considered a novel agent for CNSL, especially for relapsed/refractory patients [6,13]. In this review, we systematically searched the latest evidence of BTKis for CNSL and included 21 studies that involved 368 patients in total. For newly diagnosed CNSL patients, a pooled analysis was not performed because of the small sample size. For relapsed/refractory patients, the pooled ORR was 72% (95% CI: 64–80%). In addition, BTKi monotherapy was shown to have a pooled ORR of 60% (95% CI: 50–71%) and BTKi-based regimes had a pooled ORR of 78% (95% CI: 68–86%). This suggests that BTK combination therapy may be more effective than monotherapy. In addition, the high recurrence rate and dismal prognosis are hallmarks of CNSL. Reducing the recurrence rate and prolonging the survival time of patients with cancer is still an important issue for clinicians to consider. Therefore, we also analyzed the mPFS and mOS of patients after BTKi therapy, which showed the pooled mPFS was 5.17 months and the mOS was 10.21 months for relapsed/refractory patients.

In relevant studies of patients with newly diagnosed CNSL, the ibrutinib/HD-MTX combination could achieve an ORR of 82% (9/11), including the CR (64%) and PR (18%) [38]. For the orelabrutinib-based regimens, the ORR were 100% (one CR, one unconfirmed complete response (uCR), and two PR) [22]. However, in a clinical trial without BTKis, the ORR of MTX/cytarabine/rituximab and MTX/cytarabine regimens for newly diagnosed PCNSL were 74% and 53%, respectively [42]. In addition, patients with newly diagnosed CNSL also showed better survival with the BTKi-containing regimens. In patients with PCNSL-DLBCL who were treated with ibrutinib/rituximab/MTX or zanubrutinib/rituximab/lenalidomide/temozolomide, the mPFS was 20 months and 5 months, respectively, and the mOS was 42 months and not reached, respectively. However, in the regimens with MTX or rituximab/cyclophosphamide/liposomal doxorubicin/vincristine/prednisone, the mPFS was 7 and 1.5 months, respectively, and the mOS was 16.5 and 4.5 months, respectively. These durations were shorter than that of the regimen containing BTKis overall [37]. This demonstrates that treatment regimens containing BTKis have great potential for patients with newly diagnosed CNSL and further supports the use of BTKis in CNSL.

In addition to newly diagnosed CNSL, more studies have focused on the efficacy of BTKis for relapsed/refractory CNSL and have shown some efficacy of BTKi monotherapy, mainly including ibrutinib or tirabrutinib. In a prospective single-arm study, patients who received ibrutinib monotherapy achieved an ORR of 59% (26/44), an mPFS of 4.8 months, and an mOS of 19.2 months [30]. For patients treated with tirabrutinib monotherapy, the ORR was 63.6% (28/44), with an mPFS of 2.9 months, and the mOS was not reached [18]. A retrospective analysis showed that the use of ibrutinib monotherapy was associated with higher response rates (the ORR was 78% vs. 46%) and longer mPFS (13.1 months vs. 3 months) and mOS (16.8 months vs. 4.4 months) than chemotherapy regimens (MTX, cytarabine, and ifosfamide) [43]. Our study showed the pooled ORR, CR, and PR were 60%, 27%, and 26% for BTKi monotherapy, and the mPFS was 4.8 months (95% CI%: 2.80, 12.7) and 2.9 months (95% CI: 1.80, 11.10) in two small, simple studies with high heterogeneity [18,30].

In addition, for BTKi combination therapy in relapsed/refractory patients, our study showed the pooled ORR, CR, and PR were 78%, 48%, and 22%, respectively, and the mPFS was 5.36 months (95% CI: 4.08, 6.65). The above results suggest that BTKi combination therapy may be superior to monotherapy in the treatment of CNSL, which is similar to some other studies. BTKi combination therapy had a higher response rate and longer survival for CNSL patients compared to monotherapy [27,28,29,41]. High-dose chemotherapy followed by autologous stem cell transplantation (HDT-ASCT), ifosfamide-based immunochemotherapy, and folate antimetabolites are also effective treatment options for relapsed/refractory patients [7,44]. Among relapsed/refractory CNSL patients who received rituximab, high-dose cytarabine, and thiotepa followed by HDT-ASCT consolidation with carmustine/rituximab/thiotepa conditioning, 56% of them achieved CR [45]. In a study of relapsed/refractory CNSL patients treated with rituximab, ifosfamide, and etoposide therapy, the ORR and CR were 41% and 37%, respectively [46]. In a phase I multicenter clinical trial, patients with recurrent or progressive CNSL were treated with pemetrexed (a folate antimetabolite chemically similar to MTX), without BTKis, and the results showed that the ORR was 57.1%, and the mPFS was 4.2 months [47]. Combining the results of our study, it can be concluded that BTKis combined with chemotherapy or other regimens may have better efficacy and may prolong the survival time of patients in the treatment of relapsed/refractory CNSL, which is an effective option, especially for patients who are not suitable for chemoimmunotherapy alone.

We performed a meta-analysis of grade 3–5 AEs from regimens containing BTKis and found that the main AEs for all types of BTKis were hematological. The pooled grade 3–5 hematological AEs mainly included thrombocytopenia, neutropenia, anemia, leukopenia, lymphopenia, and febrile neutropenia, with an incidence of 4–19%. In addition to the hematological AEs, the highest incidence was infection (12%). The results of our research also recommend that doctors should focus on the prevention of AEs, such as atrial fibrillation, bleeding, aspergillosis, and transaminase increase, although the incidence was 1–5%. For ibrutinib, the most common AE was lymphopenia (19%). Furthermore, the rates of infection, thrombocytopenia, and transaminase increase were 14%, 13%, and 13%, respectively. The second-generation BTKis mainly included neutropenia, leukopenia, thrombocytopenia, infection, bleeding, and transaminase increase. Among these, neutropenia was the most common (11%). The common AEs in regimens containing orelabrutinib included leukopenia, fatigue, increased dehydrogenase, erythrocytopenia, and decreased hemoglobin. And the grade ≥ 3 AEs mainly included thrombocytopenia, leukopenia, decreased hemoglobin, and fatigue [21,22]. No atrial fibrillation and hemorrhage have been reported. In the treatment regimen of tirabrutinib, neutropenia was the most common grade ≥ 3 AE (9.1%), and no cardiovascular-related AEs were observed [18]. Yoshioka et al. showed that none of the five patients studied had serious AEs [26]. For the zanubrutinib combination regimen, the most common grade 3–4 AE was also neutropenia (38%); no bleeding or cardiac events happened while the patients were receiving treatment [19]. In general, although BTKis developed grade ≥ 3 AEs, the incidence was low and generally manageable, demonstrating the safety and effectiveness. Among the AEs of special interest for BTKis, the incidence of bleeding was 2% for both ibrutinib and the second-generation BTKis. However, the incidence rates of infection were 14% for ibrutinib and 3% for second-generation BTKis. In addition, atrial fibrillation was not observed in the second-generation BTKis, suggesting that the second-generation BTKis displayed a better safety profile than the first generation.

Although the data supporting BTKis in the treatment of CNSL have been previously reviewed, our study collected the most recent evidence. The existing reviews have mainly focused on relapsed/refractory CNSL patients or PCNSL patients, while our review performed the analysis for newly diagnosed CNSL as well as the relapsed/refractory CNSL, which can make the evidence more comprehensive. In addition, although we have come to a comprehensive result for BTKis, there are still several limitations. We did not perform a quantitative publication bias detection by Egger’s and Begg’s tests, but potential publication bias may still exist because of a lack of controlled groups and small sample sizes in the included studies. Therefore, more prospective studies, especially well-designed RCTs are still needed. Furthermore, we only included 35 newly diagnosed patients in this review, and more data are still needed for BTKis as a first-line treatment for CNSL.

## 5. Conclusions

According to the current evidence, we found that BTKis could be considered as an effective and safe treatment for relapsed/refractory CNSL, as well as newly diagnosed CNSL. Due to the potential bias of the evidence, it may lead to misunderstanding and instability in this review. In the future, large and well-designed trials are still needed to confirm the promising treatment of BTKis in CNSL.

## Figures and Tables

**Table 1:** Information of the included studies.

No. Study		Country	Study Design	No. of Patients	Male/ Female	Median (Range) Age, Years	CNSL Status	Type of CNSL	Intervention	Outcomes				
										ORR (%)	CR (%)	PR (%)	mPFS (Month)	mOS (Month)
1	Chamoun K 2016 [31]	US	Retrospective case series	14	9/5	68 (48–79)	Relapsed/refractory	Primary	Ibrutinib monotherapy	50	21	29	-	-
2	Grommes C 2017 [32]	US	Prospective	20	8/12	69 (21–85)	Relapsed/refractory	Primary/secondary	Ibrutinib monotherapy	70	45	25	4.6	-
3	Lionakis MS 2017 [33]	US	Retrospective	18	11/70	66 (49–87)	Newly diagnosed and relapsed/refractory	Primary	Ibrutinib/DA-TEDDi-R combination	93	86	7	15.3	NR
4	Soussain C 2019 [30]	France	Prospective	52	24/28	67.5 (47–82)	Relapsed/refractory	Primary	Ibrutinib monotherapy	52	19	33	4.8	19.2
5	Grommes C 2019 [35]	US	Prospective	15	8/7	62 (23–74)	Newly diagnosed and relapsed/refractory	Primary/secondary	Ibrutinib-based combination therapy	80	53	27	9.2	NR
6	Chen F 2020 [38]	China	Retrospective	11	7/4	56 (41–68)	Newly diagnosed	Primary	Ibrutinib/MTX combination	82	64	18	7.4	NR
7	Lauer EM 2020 [28]	UK	Retrospective	9	NA	63 (53–82)	Relapsed/refractory	Primary/secondary	Ibrutinib monotherapy or in combination with other regimens	66	66	0	9.2	NR
8	Chen YD 2020 [40]	China	Retrospective	18	10/8	58.5 (18–76)	Relapsed/refractory	Primary	Ibrutinib-based regimen	83	55	28	6	15
9	Lewis KL 2020 [27]	Australia	Prospective	33	23/10	64 (22–85)	Relapsed/refractory	Primary/secondary	Ibrutinib monotherapy or in combination with other regimens	58	55	3	-	-
10	Narita Y 2020 [18]	Japan	Prospective	44	24/20	60 (29–86)	Relapsed/refractory	Primary	Tirabrutinib	64	34	30	2.9	NR
11	Dalma DM 2021 [29]	Romania	Retrospective case series	3	1/2	60 (53–64)	Relapsed/refractory	Primary	Ibrutinib monotherapy or in combination with other regimens	67	67	0	-	-
12	Yu HF 2021 [9]	China	Retrospective	3	1/2	76 (45–79)	Newly diagnosed and relapsed/refractory	Primary	Ibrutinib monotherapy or in combination with other regimens	100	67	33	-	-
13	Zhang Y 2021 [19]	China	Retrospective case series	13	3/10	53 (52–69)	Newly diagnosed and relapsed/refractory	Primary	Zanubrutinib-based regimens	88	88	0	-	-
14	Renaud L 2021 [36]	France	Retrospective	22	12/10	71 (44–89)	Relapsed/refractory	Primary/secondary	Ibrutinib and temozolomide	55	14	41	5.3	8.9
15	Song J 2021 [37]	China	Retrospective	49	32/17	63 (33–81)	Newly diagnosed	Primary	ibrutinib or zanubrutinib in combination with other regimens	-	-	-	i: 20 z: 5	i: 42 z: NR
16	Yoshioka H 2022 [26]	Japan	Retrospective case series	5	1/4	76 (62–77)	Relapsed/refractory	Primary	Tirabrutinib-based regimens	100	0	100	-	-
17	Wu JJ 2022 [22]	China	Retrospective	23	15/8	55 ± 13.78 (mean)	Newly diagnosed and relapsed/refractory	Primary/secondary	Orelabrutinib monotherapy or orelabrutinib-based regimens	68	31	37	-	-
18	Yang C 2022 [21]	China	Retrospective	15	5/10	62 (33–78)	Relapsed/refractory	Primary	Combination of rituximab, HD-MTX, temozolomide, orelabrutinib, and lenalidomide	86	73	13	9.8	NR
19	Guan WX 2022 [39]	China	Prospective	10	3/7	52 (41–74)	Newly diagnosed and relapsed/refractory	Primary	BTKi (ibrutinib zanubrutinib or orelabrutinib) monotherapy treatment	90	70	20	-	-
20	Wang WH 2022 [41]	China	Retrospective	43	22/21	53 (52–69)	Relapsed/refractory	Primary/Secondary	Ibrutinib-based regimens	74	18	56	-	-
21	Wang S 2023 [34]	China	Retrospective	14	10/4	58 (37–80)	Relapsed/refractory	Primary/Secondary	Ibrutinib-based regimens	78	57	21	4	-

NA = not applicable; NR = not reached; i = ibrutinib; z = zanubrutinib.

**Table 2:** JBI Critical Appraisal Checklist of Case Series for included studies.

Studies	Q1	Q2	Q3	Q4	Q5	Q6	Q7	Q8	Q9	Q10
Chamoun K 2016 [31]	Yes	No	Unclear	Unclear	Yes	No	Yes	Yes	No	NA
Grommes C 2017 [32]	Yes	Yes	Unclear	Unclear	Unclear	No	Yes	Yes	No	Yes
Lionakis MS 2017 [33]	Yes	No	Unclear	Unclear	No	No	Yes	Yes	No	Yes
Soussain C 2019 [30]	Yes	Yes	Unclear	Yes	Yes	No	Yes	Yes	No	Yes
Grommes C 2019 [35]	Yes	No	Unclear	Unclear	No	No	Yes	Yes	No	Yes
Chen F 2020 [38]	Yes	Yes	Unclear	No	No	No	Yes	Yes	No	Yes
Lewis KL 2020 [27]	Yes	Yes	Unclear	Unclear	Yes	No	Yes	Yes	No	Yes
Lauer EM 2020 [28]	Yes	Yes	Yes	No	No	No	Yes	Yes	No	Yes
Narita Y 2020 [18]	Yes	No	Unclear	Unclear	Yes	No	Yes	Yes	No	Yes
Chen YD 2020 [40]	Yes	No	Unclear	No	No	No	Yes	Yes	No	Yes
Dalma DM 2021 [29]	Yes	No	Unclear	No	No	No	Yes	Yes	No	NA
Renaud L 2021 [36]	Yes	Yes	Yes	Yes	Yes	No	Yes	Yes	No	Yes
Yoshioka H 2021 [26]	Yes	No	Unclear	Unclear	No	No	Yes	Yes	No	NA
Zhang Y 2021 [19]	Yes	No	Unclear	Unclear	No	No	Yes	Yes	No	Yes
Yu HF 2021 [9]	Yes	No	Unclear	Yes	No	No	Yes	Yes	No	NA
Song J 2021 [37]	Yes	No	Unclear	Unclear	No	No	Yes	Yes	No	Yes
Yang C 2022 [21]	Yes	Yes	Yes	Yes	No	No	Yes	Yes	No	Yes
Wu JJ 2022 [22]	Yes	Yes	Yes	Yes	No	No	Yes	Yes	No	Yes
Guan WX 2022 [39]	Yes	Yes	Yes	Yes	No	No	Yes	Yes	No	Yes
Wang WH 2022 [41]	Yes	No	Unclear	Unclear	No	No	Yes	Yes	No	Yes
Wang S 2023 [34]	Yes	Yes	Unclear	Unclear	No	No	Yes	Yes	No	Yes

NA = not applicable.

**Table 3:** Quantitative description of newly diagnosed CNSL.

Study	ORR					CR					PR				
	Event Number	Total	Effect Size	Lower CI	Upper CI	Event Number	Total	Effect Size	Lower CI	Upper CI	Event Number	Total	Effect Size	Lower CI	Upper CI
Chen F [38]	9	11	0.82	0.52	0.95	7	11	0.64	0.35	0.85	2	11	0.18	0.05	0.48
Wu JJ [22]	4	4	1.00	0.51	1.00	2	4	0.50	0.15	0.85	2	4	0.50	0.15	0.85
Guan WX [39]	6	6	1.00	0.61	1.00	4	6	0.67	0.30	0.90	2	6	0.33	0.10	0.70
Zhang Y [19]	4	4	1.00	0.51	1.00	4	4	1.00	0.51	1.00	0	4	0.00	0.00	0.49
Yu HF [9]	2	2	1.00	0.34	1.00	1	2	0.50	0.09	0.91	1	2	0.50	0.09	0.91
Lionakis MS [33]	5	5	1.00	0.57	1.00	0	5	0.00	0.00	0.43	5	5	1.00	0.57	1.00
Grommes C [35]	3	3	1.00	0.44	1.00	1	3	0.53	0.23	0.82	2	3	0.43	0.13	0.76

**Table 4:** Grade 3–5 AEs of BTKis.

Adverse Event in Detail	All Types of BTKis (95% CI)	I^2^	Ibrutinib (95% CI)	I^2^	Second-Generation BTKis (95% CI)	I^2^
Thrombocytopenia	0.09 (0.02, 0.18)	55.82%	0.13 (0.07, 0.21)	0.00%	0.04 (0.00, 0.19)	68.06%
Neutropenia	0.12 (0.04, 0.22)	67.96%	0.12 (0.03, 0.26)	68.64%	0.11 (0.00, 0.34)	77.83%
Anemia	0.12 (0.05, 0.20)	0.00%	0.12 (0.05, 0.20)	0.00%	NA	
Leukopenia	0.10 (0.02, 0.20)	74.96%	0.10 (0.01, 0.25)	78.61%	0.09 (0.00, 0.30)	76.17%
Lymphopenia	0.19 (0.00, 0.56)	92.37%	0.19 (0.00, 0.56)	92.37%	NA	
Febrile neutropenia	0.04 (0.00, 0.19)	53.85%	0.04 (0.00, 0.19)	53.85%	NA	
Aspergillosis	0.03 (0.00, 0.8)	0.00%	0.03 (0.00, 0.8)	0.00%	NA	
Infection	0.12 (0.04, 0.22)	72.33%	0.14 (0.05, 0.27)	75.07%	0.03 (0.00, 0.10)	0.00%
Bleeding	0.02 (0.00, 0.06)	0.00%	0.02 (0.00, 0.06)	0.00%	0.02 (0.00, 0.14)	0.00%
Atrial fibrillation	0.01 (0.00, 0.04)	0.00%	0.01 (0.00, 0.04)	0.00%	NA	
Transaminase increase	0.05 (0.00, 0.14)	66.13%	0.13 (0.01, 0.31)	75.96%	0.01 (0.0, 0.06)	0.00%

NA = not applicable.

## Data Availability

The data generated in this study are included in this article and its Supplementary Materials. Additional enquiries are available from the corresponding authors upon request.

## Supplementary Materials

The following supporting information can be downloaded at: https://www.mdpi.com/article/10.3390/cancers16050860/s1, S1: Detailed PRISMA checklist. S2: Detailed search strategy. S3: Any grade AEs of BTKis.
